# Commentary: Musicians' Online Performance during Auditory and Visual Statistical Learning Tasks

**DOI:** 10.3389/fnhum.2017.00603

**Published:** 2017-12-11

**Authors:** Federica Menchinelli, Petra M. J. Pollux, Simon J. Durrant

**Affiliations:** Psychology, University of Lincoln, Lincoln, United Kingdom

**Keywords:** auditory statistical learning, visual statistical learning, ERPs (Event-Related Potentials), triplet learning, musicians and non-musicians

Statistical learning (SL) is the extraction of the underlying statistical structure from sensory input (Frost et al., 2015). The extent to which this ability is domain-general (with a single central mechanism underpinning SL in any modality) or domain-specific (where the SL mechanism differs by modality) remains a central question in statistical learning (Frost et al., 2015), and two approaches have been adopted to tackle this. First is to examine the extent to which predominantly domain-specific skills such as language proficiency (Arciuli and von Koss Torkildsen, 2012) and musical expertise (Schön and François, 2011), and domain-general skills such as working memory and general IQ (Siegelman and Frost, 2015), correlate with SL ability. Second is to compare SL performance across modalities, or even examine cross-modal transfer (Durrant et al., 2016).

Mandikal Vasuki et al. (2017) (and the sister paper: Mandikal Vasuki et al., 2016) make an important contribution by adopting both of these approaches. They compare auditory and visual SL using the Saffran triplet learning paradigm (Saffran et al., 1999) in musicians and non-musicians. The three key findings are that musicians are better than non-musicians at segmentation of auditory stimuli only, there is no correlation between auditory and visual performance, and that auditory performance is better overall. This last result could be due to privileged auditory processing of sequential stimuli (Conway et al., 2009), or it could just reflect differences in perceptual or memory capabilities across modalities. However, the fact that SL performance in one modality does not predict performance in another is hard to explain if a single mechanism underlying both is posited. Combined with the fact that overall better performance was found in musicians only in the auditory modality, a domain-specific SL mechanism seems to offer the most parsimonious explanation of this data.

One of the key strengths of this study is the unusual choice to record ERPs. Behavioral measures of learning during passive exposure are problematic—especially if the nature of the stimuli is to remain hidden from participants—so ERP recording allows online measurement of learning performance during exposure, and provides insight into the underlying mechanism. In keeping with the behavioral results, differences in the N1 and N400 triplet onset effects between musicians and non-musicians were seen only for the auditory stimuli, while the N400 was not seen at all for visual stimuli. These could suggests a neural mechanism for auditory statistical learning different to that of visual statistical learning, but without source localization based on more electrodes, this remains speculative.

ERP data also provides insight into the time course of learning. Thanks to this method we know that an advantage of musicians in auditory SL is that they are “fast learners”; they begin segmentation of the stimulus stream from earlier in the exposure. This difference could not have been detected behaviorally. It would have been interesting to also see the difference in ERP responses to correct and incorrect triplets in the behavioral task and this is certainly worth including in future reports. In addition, there are large individual differences in SL (Siegelman and Frost, 2015), hence ERPs of participants with widely varying performance is therefore potentially of great interest and exclusion based on behavior should be limited. In the present study, only a small number of participants were excluded so this was not a major problem, but in future ERP studies we would caution against the use of the relatively narrow outlier exclusion criteria (±2 SD) seen here.

The present study offers into statistical learning across modalities, but key questions remain, including the fidelity of SL (how accurately are specific transition probabilities learned) and the order of SL (can higher-order transitions be effectively learned in a short exposure). The triplet learning paradigm is unable to provide insight into either of those questions because it mixes first- and second-order transitions and does not sample a range of probabilities. Other approaches such as the transition matrix paradigm (Durrant et al., 2011), by allowing precise control of the transition order and the transition probabilities, may be more suitable to answer these questions, especially if combined with ERP measurements.

Another important limitation of the triplet paradigm, which is particularly relevant for this study, is the role of prior preferences for particular triplets. Probably all participants will have had extensive exposure to Western tonal music, which results in the development of cognitive schemata (Krumhansl, 1990) reflecting tone distribution statistics in Western tonal music (Knophoff and Hutchinson, 1983). These are acquired in early childhood through passive exposure (Speer and Meeks, 1985), and generate expectations of tones in a sequence (Bharucha, 1994). Saffran et al. (1999) attempted to counteract this by using a two-language crossover design and avoiding stereotypical patterns within the triplets. Their results showed a preference for particular triplets within both languages which may reflect prior exposure to Western tonal music and which is much stronger than the effect of short-term exposure within the experiment (Hazan et al., 2008). The present study used only Saffran's Language 1, and these triplet preferences based on prior musical exposure might explain the difference between musicians and non-musicians in the auditory domain. Future studies should ideally measure prior preference of triplets and potentially try to control them through the use of non-Western scales such as the Bohlen-Pierce scale (Durrant et al., 2011).

Combining auditory and visual SL with a comparison of musicians and non-musicians is the main contribution of this paper. The results of this study may be interpreted as evidence of a domain-specific component to SL in keeping with other findings (Conway and Christiansen, 2006) but alternative accounts suggest that a domain-general component is equally possible (Thiessen, 2011). Future investigations could use more sophisticated instruments such as the Gold MSI (Müllensiefen et al., 2014), to look for effects on specific subscales of musical experience, to better understand why musicians have an advantage in the auditory modality in particular. The present study is an important first step toward this.

## Author contributions

FM, PP, and SD conceived the ideas in the article, discussed the specific arguments to be presented, and wrote the manuscript.

### Conflict of interest statement

The authors declare that the research was conducted in the absence of any commercial or financial relationships that could be construed as a potential conflict of interest.
